# Prooxidant Effects of Epigallocatechin-3-Gallate in Health Benefits and Potential Adverse Effect

**DOI:** 10.1155/2020/9723686

**Published:** 2020-08-12

**Authors:** Jie Ouyang, Kun Zhu, Zhonghua Liu, Jianan Huang

**Affiliations:** ^1^National Research Center of Engineering Technology for Utilization of Functional Ingredients from Botanicals, Hunan Agricultural University, Changsha, China; ^2^Collaborative Innovation Centre of Utilization of Functional Ingredients from Botanicals, Hunan Agricultural University, Changsha, China; ^3^Key Laboratory of Tea Science of Ministry of Education, Hunan Agricultural University, Changsha, China

## Abstract

Epigallocatechin-3-gallate (EGCG) is the major polyphenolic compound present in green tea and is generally regarded as an effective antioxidant. However, its chemical reactivity makes it susceptible to generate reactive oxygen species (ROS) via autooxidation and exhibit prooxidant effects. The prooxidant actions of EGCG could play a dual role, being both beneficial and harmful. This review summarized recent research progress on (1) the anticancer, antiobesity, and antibacterial effects of EGCG and (2) the possible toxicity of EGCG. The major focus is on the involvement of prooxidant effects of EGCG and their effective doses used. Considering dosage is a crucial factor in the prooxidant effects of EGCG; further studies are required to find the appropriate dose at which EGCG could bring more health benefits with lower toxicity.

## 1. Introduction

Tea is the second most popular beverage consumed in the world, next to water. Green tea is a kind of nonfermented tea produced from the plant *Camellia sinensis*. It is favored by people for its fresh flavor and health benefits and consumed worldwide, especially in East Asian countries.

Green tea contains caffeine and polyphenolic compounds known as catechins. Catechins are the primary bioactive substances and present significant biological properties. Catechins constitute up to 30-40% of tea leaves' dry weight, among that EGCG is the main and the most abundant catechin [1, 2]. EGCG has been traditionally regarded as beneficial, mainly ascribed to its antioxidant action [3]. The antioxidant effects of EGCG are manifested in scavenging free radicals in the body and inhibiting the formation of ROS [4]. The results of earlier studies suggested that EGCG could decrease the risk of several human disorders associated with oxidative stress [5].

On the other hand, EGCG also displays significant prooxidant effects, usually under high-dose conditions. The prooxidant actions of EGCG play a dual role, being both beneficial and harmful. While achieving desired outcomes in chronic disease prevention and treatment, reports about the toxicity of EGCG are also emerging [6]. A growing body of evidence continues to demonstrate a variety of harmful effects from excessive consumption of green tea or oral administration of high-dose EGCG supplement [7]. High doses of EGCG not only cause cytotoxicity in vitro but also result in living body hepatotoxicity, nephrotoxicity, and gastrointestinal disorders (vomiting and diarrhea) [6].

The oral bioavailability of EGCG is not so profound in healthy humans as it was only 0.2-2% of the total ingestion [8]. Most of the ingested EGCG is absorbed in the small intestine and substantially degraded in the large intestine by microbiota action [9]. The effective dosage of EGCG might be close to or higher than the toxic dosage in practical applications, considering its low bioavailability. Therefore, it is necessary to understand the potential toxicity, doses, and usage of EGCG. In this review, the prooxidant effects of EGCG in health benefits and adverse effects were discussed, especially concerning their underlying mechanisms involved and doses used. This review is aimed at harnessing the prooxidant effects of EGCG for human health maintenance while avoiding toxicity, thereby better guiding the safety consumption of green tea and EGCG.

## 2. Chemical Structure and Autooxidation of EGCG

**Figure 1:**
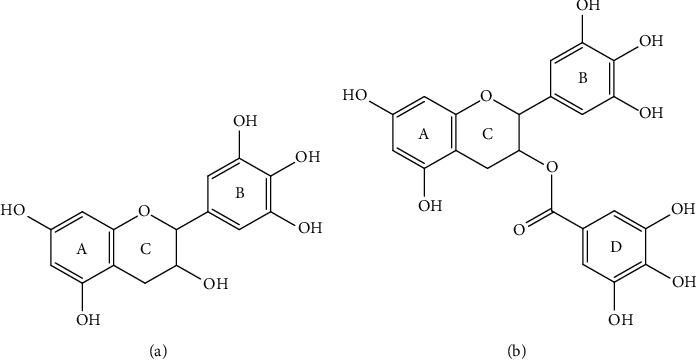
(a) Basic structure of catechins. (b) Chemical structure of EGCG.

Basic catechins contain two or more aromatic rings. Hydroxyl group on carbon three position and/or the higher degree of hydroxylation of the B ring would be primarily responsible for the potent antioxidant activities of catechins (Figure 1(a)) [10]. Previous structure-activity relationship studies of catechins have demonstrated the importance of the number and location of the phenolic hydroxyl groups on antioxidative capacity [1]. EGCG has the remarkable potential to scavenge radicals and chelate metal ion. These abilities could be ascribed to the presence of dihydroxy and trihydroxy groups in A ring, B ring, and D ring (Figure 1(b)) [11].

**Figure 2:**
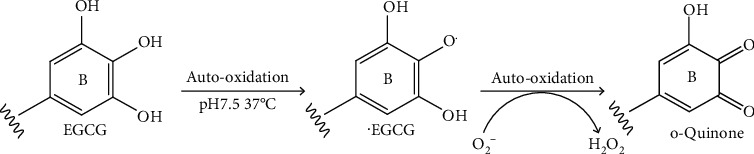
Superoxide-mediated chain reaction: the formation of o-quinone.

The catechol structure of EGCG makes it susceptible to degradation via autooxidation (Figure 2). Under normal physiological conditions (pH 7.4, 37°C), EGCG is autooxidized and converted to o-quinone through nonenzymatical dehydrogenation of phenolic hydroxyl groups at B ring [12]. When the cell culture medium is exposed in the air, EGCG could be oxidized by oxygen and yields superoxide anion radicals (O_2_^−^) and EGCG radicals (^·^EGCG). O_2_^−^ and ^·^EGCG are essential intermediate products in EGCG autooxidation. O_2_^−^ and oxygen could function as oxidants for further oxidation of EGCG, finally resulting in the formation of o-quinone, accompanying the generation of hydrogen peroxide (H_2_O_2_). O_2_^−^ could also form substantial amounts of H_2_O_2_ via disproportionation reaction [13]. One EGCG molecule could produce more than two H_2_O_2_ molecules in phosphate buffer at neutral pH [14].

Autooxidation of EGCG generates substantial ROS. The ROS comprises singlet oxygen, hydroxyl radicals, superoxide, peroxides, and H_2_O_2_. H_2_O_2_ is in a dominant position and usually is regarded as a toxic agent. When the ROS level exceeds cellular antioxidant capacity, oxidative stress will occur. In other words, this is the result of an imbalance between prooxidant and antioxidant effects. Inclusion of antioxidant defense enzymes such as catalase (CAT) and superoxide dismutase (SOD) could minimize H_2_O_2_ level, which is essential to maintain the redox balance.

The concentration of EGCG in the cell environment seems to be a primary factor in explaining its prooxidant effects [15]. For example, EGCG treatment alone diminished DNA strand breakage of human blood lymphocytes at low concentrations (0.1-0.01 *μ*M), while it induced DNA strand breakage at higher concentration (1-100 *μ*M) [16]. Thus, EGCG acts as an effective antioxidant at low doses (within the range of high nanomolar and low micromolar levels), while EGCG represents a prooxidant at high doses. However, this blurred boundary might vary depending on the type of radical stimulants, cellular environment, and duration of exposure to EGCG [17].

## 3. Health Benefits

Until now, EGCG has been a major research subject within the field of health-promoting effects. The potential role of the prooxidant effects of EGCG in cancer and obesity prevention and treatment, as well as the antibacterial actions, achieved demonstrable results in previous studies.

### 3.1. Prooxidant Effects and Anticancer Activity of EGCG

Cancer is one of the most common and life-threatening diseases occurring among humankind. EGCG, as a natural product, has drawn a great deal of attention from both the scientific community and the general public. Indeed, EGCG has shown both prophylactic and therapeutic efficacy in multiple human cancers. Several mechanisms have been proposed to account for the inhibitory action of EGCG against cancer formation and growth. The prooxidant effects of EGCG were thought to be potential mechanisms for anticancer action. The anticancer mechanisms varied depending on the cell type, dose, and/or time of treatment (Table 1) [18–36].

Apoptosis is the best-described form of programmed cell death. The induction of apoptosis represents a universal and ideal therapeutic strategy for cancer control. Cell apoptosis could be triggered by either the intrinsic mitochondrial pathway or the external death receptor pathway [37]. The mitochondrial pathway could be induced by intracellular stresses, such as oxidative stress.

The apoptosis-triggering effects of ROS have been noted in vitro (Table 1). EGCG inhibited cell growth in a dose-dependent manner, and the decrease in the number of viable cells was mainly due to apoptosis caused by the EGCG-induced intracellular ROS. As early as the last century, scientists found that EGCG induced H_2_O_2_ formation in human lung cancer cell lines H661 and 21BES, and exogenously added CAT completely prevented EGCG-induced cell apoptosis, which suggested that H_2_O_2_ is involved in the apoptosis process provoked by EGCG [27]. Similar actions were also found in various cancers and tumor cells (Table 1). Thioredoxin (Trx) and thioredoxin reductase (TrxR) are pivotal regulators of cellular redox homeostasis. Decreased Trx/TrxR activity might contribute to the increased ROS level. High concentration of EGCG inactivated Trx/TrxR via the formation of EGCG-Trx1 and EGCG-TrxR conjugates, which was linked to the elevation of ROS level in HeLa cells, and further promoted cancer cell death [22]. Moreover, one of the biochemical hallmarks of apoptosis is genomic DNA fragmentation. Chen et al. performed the DNA fragmentation assay in the SKOV-3 cells, indicating that EGCG induced apoptosis by causing DNA damage [18]. This result was consistent with other studies in ovarian and cervical cancer cells [21, 32].

In terms of molecular mechanisms, intrinsic apoptosis leads to the release of mitochondrial cytochrome c. After being released into the cytoplasm, cytochrome c stimulates apoptosome formation, followed by activation of caspase cascades [37]. EGCG-mediated mitochondrial ROS could promote cytochrome c release to the cytosol. The antiproliferative action of EGCG on prostate cancer and breast cancer is mediated through apoptosis as evident from caspase-9 [19, 36]. The cells susceptible to EGCG-induced apoptosis also showed activation of caspase-3 [38]. Moreover, the increased ROS level was observed to result in the stimulation of mitogen-activated protein kinase (MAPK) [39]. The MAPK signaling pathway, including extracellular signal-regulated kinase (ERK), Jun N-terminal kinase (JNKs), and p38, plays a vital contribution in cell proliferation, differentiation, apoptosis, and stress response. EGCG induced oxidative stress via generation of ROS and thereafter activated the JNK pathway, leading to changes in mitochondrial membrane potential and release of cytochrome c in HT-29 human colon adenocarcinoma cells and MIA PaCa-2 pancreatic cancer cells [24, 34]. Together, these results suggest that EGCG-induced apoptosis is mediated through ROS generation and might subsequently activate the cell intrinsic pathway [33].

In the presence of transition metals such as copper and iron, H_2_O_2_ could convert to a potent oxidant hydroxyl radical via the Fenton reaction. Nakagawa et al. found that EGCG (12.5-50 *μ*M) produced H_2_O_2_ and triggered Fenton reaction to form highly toxic hydroxyl radicals, which resulted in lymphoblastic leukemia Jurkat cell death [29]. In the presence of Fe(III) and Cu(II), EGCG (5-20 *μ*M) induced DNA damage in HL-60 cells, as 8-oxo-7,8-dihydro-2′-deoxyguanosine (8-oxodG) content increased, which was a characteristic of oxidative DNA damage [40]. Nevertheless, no significant increase in 8-oxodG was observed in H_2_O_2_-resistant colon HP100 cells, suggesting that H_2_O_2_ was involved in cellular DNA damage. EGCG could inhibit cell proliferation and induce apoptosis through cellular DNA breakage in different cancer cell lines [41]. Such DNA breakage involved the mobilization of endogenous copper ions and the generation of ROS. Moreover, the observation of site specificity of DNA damage by EGCG is valuable. Cu(II)-mediated DNA damage by EGCG occurred most frequently at T and G residues [40]. EGCG was able to mobilize endogenous copper ions and generate hydroxyl radicals in situ [42]. Hydroxyl radicals served as the proximal DNA cleaving agent, leading to DNA breakage in the nuclei. This result was possibly due to the interaction of EGCG with chromatin-bound copper ions, and then, the nondiffusible hydroxyl radicals were formed at the binding site. Hence, hydroxyl radical generated nearby DNA was well established as the cause of strand scission. Because the concentration of copper is significantly very high in various malignancies, EGCG could induce cancer cell death through the metal ion-dependent pathway [41]. This pathway was independent of mitochondria-mediated programmed cell deaths. Such action involved in metal ion-mediated DNA cleavage would be an important mechanism of anticancer properties of EGCG.

In addition to being transported into the cell, EGCG could also function on the cell membrane fraction to regulate the surface growth factor receptor [43]. Earlier studies found that autooxidation of EGCG led to epidermal growth factor receptor (EGFR) inactivation in human esophageal cancer cell line KYSE 150 [26]. One possible explanation is that H_2_O_2_ produced from EGCG autooxidation in the cell culture medium could attack and inactivate EGFR, leading to the inhibition of EGFR phosphorylation.

It is worth considering whether high amounts of EGCG could cause damage to normal cells. EGCG-mediated ROS production was particularly observed in cancer cells compared with normal cells. The selectivity of EGCG-induced apoptosis in cancer cells might be due to the differential inducibility of ROS and preferential expression of apoptosis-related genes [23]. Moreover, Tao et al. found that EGCG induced differential mitochondrial dysfunction and oxidative stress in normal and oral cancer cells. These effects were related to the differential modulation of sirtuin 3 (SIRT3) and its downstream targets, including glutathione (GSH) and SOD [31]. Considering the cytotoxicity of EGCG in normal cells, the IC_50_ value in normal cells was checked and showed to be more than 200 *μ*M, while that for the corresponding cancer cells was 132 *μ*M [25]. These results suggested that cancer cells are more sensitive to EGCG than normal cells, and ROS might be selectively toxic to cancer cells.

In addition to being used as preventive agents individually, EGCG could also be used as adjuvant therapies. Generally, cooperative interaction of two or more agents could target more signaling pathways, thus effectively improving agent chemosensitivity, reducing untoward effects of treatment, expanding the scope of action, and showing higher therapeutic outcomes [44]. Drug resistance is a daunting challenge in cancers. Prooxidant activities of EGCG were proposed to contribute to overcoming drug resistance, highlighted by the fact that H_2_O_2_ production induced by EGCG increased the potency of cisplatin in ovarian cancer cells by three to sixfold [45]. In contrast, cisplatin alone was highly resistant to the treatment in some cancer cell lines. Copper transporter 1 (CTR1) is a critical determinant to increase cisplatin uptake. EGCG could upregulate CTR1 expression through the stimulation of ROS [39]. Simultaneous treatment of arsenic trioxide (ATO) with EGCG showed oxidative-mediated induction of apoptosis in leukemia cancer cells [46]. EGCG acted as a prooxidant and increased intracellular H_2_O_2_, and ATO-induced heme oxygenase-1 (HO-1) provided ferrous iron to increase the Fenton reaction. In both cases, cellular oxidative damage eventually occurred.

**Figure 3:**
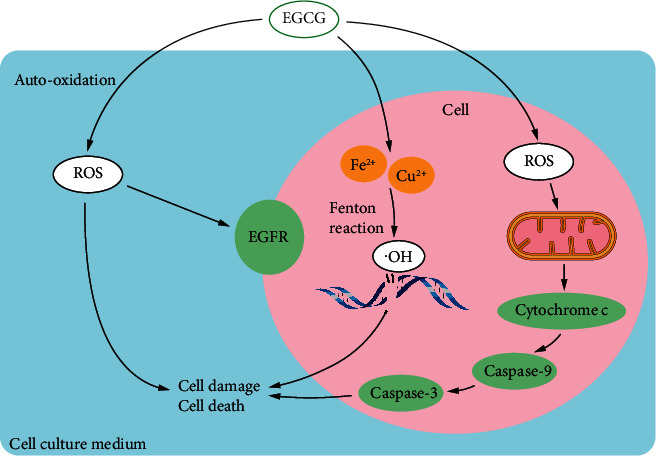
Prooxidant effects of EGCG in cell culture.

In general, under typical cell culture conditions, EGCG has been known to generate (i) extracellular ROS via autooxidative reaction in the cell culture medium, (ii) ROS in cellular mitochondria, and (iii) intracellular ROS through the Fenton reaction upon cell entry (Figure 3). These three pathways contribute differently to cancer cells but eventually cause cell damage and death. Cancer initiation and progression are generally divided into several stages. When EGCG acts as an antioxidant, it might more effectively enhance antioxidant capacity at the cancer prevention stage, whereas when EGCG acts as a prooxidant it might be more critical at suppressing tumor growth stage. One possible supposition is that tumor cells may be more susceptible to oxidative stress, because their increased growth rate and metabolism cause a heightened basal ROS level. The degree of cell proliferation and differentiation seems to be one factor affecting the ROS production ability of EGCG. Future research will be required to determine if EGCG is a much more potent ROS inducer in differentiated than in undifferentiated cancer cells. Although a limited amount of data has shown that these prooxidant effects can occur in vivo, it is essential to understand when and to what extent the antioxidant or prooxidant effects of EGCG are working in different stages of cancers in animal models.

### 3.2. Prooxidant and Antiobesity Effects of EGCG

Obesity is a metabolic disease characterized by abnormal or excessive fat accumulation. It is generally associated with an increased risk of chronic diseases, including diabetes, hypertension, and dyslipidemia [10]. A large and growing body of studies has investigated the antiobesity effects of EGCG in cellular and animal experiments and the underlying mechanisms.

The clinical manifestations of obesity are adipocyte hyperplasia and hypertrophy. In vitro studies have well demonstrated that EGCG could inhibit adipocyte growth and induce adipocyte death through its prooxidant effects. Hung et al. reported that high concentrations of EGCG (50-400 *μ*M) reduced the cell viability of preadipocytes by 15-30%, induced the appearance of DNA fragmentation, and increased the activity of the apoptotic enzyme caspase-3 [47]. EGCG was demonstrated to raise ROS level and descend GSH level in preadipocytes and adipocytes, which induced oxidative stress thus resulting in decreased cell number [48].

5′AMP-regulated protein kinase (AMPK) represents a metabolite-sensing protein kinase. Hwang et al. (2005) found that the release of ROS by EGCG stimulation could further activate AMPK rapidly in 3T3-L1 adipocytes. A recent study also proved that AMPK was activated by exogenous H_2_O_2_, and this activation was not through direct redox signaling to AMPK, but was a secondary consequence of redox effects on other processes [49].

**Figure 4:**
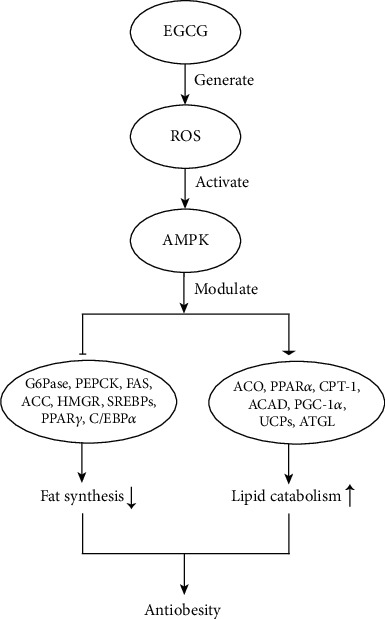
Effects of EGCG on lipid metabolism via ROS and AMPK.

EGCG activates AMPK via the generation of ROS, subsequently blocks anabolic pathways and promotes the catabolic pathway, and suppresses gluconeogenesis and adipogenesis, consequently leading to body weight reduction and metabolic syndrome alleviation (Figure 4). The activation of AMPK modulates the expression of genes and proteins involved in lipid metabolism, downregulates the expression of fat synthesis proteins, and upregulates lipid catabolism proteins [2]. It was shown that EGCG inhibited the expressions of glucose 6-phosphatase (G6Pase, for gluconeogenesis), phosphoenolpyruvate carboxykinase, (PEPCK, for gluconeogenesis), fatty acid synthase (FAS, for fatty acid synthesis), acetyl-CoA carboxylase (ACC, for fatty acid synthesis), hydroxymethylglutaryl-CoA reductase (HMGR, for cholesterol), sterol regulatory element-binding proteins (SREBPs, for sterol synthesis), peroxisome proliferator-activated receptor gamma (PPAR*γ*, for lipid synthesis and storage), and CCAAT/enhancer-binding protein alpha (C/EBP*α*, for adipogenesis) as well as enhanced the expression of acyl-CoA oxidase (ACO, for fatty acid oxidation), peroxisome proliferator-activated receptor alpha (PPAR*α*, for fatty acid oxidation), carnitine palmitoyltransferase-1 (CPT-1, for fatty acid oxidation), acyl-CoA dehydrogenase (ACAD, for fatty acid oxidation), peroxisome proliferator-activated receptor gamma coactivator-1*α* (PGC-1*α*, for fatty acid oxidation), uncoupling proteins (UCPs, for thermogenesis), and adipose triglyceride lipase (ATGL, for triglyceride hydrolysis) [50–54].

Accordingly, the prooxidant effects of EGCG play a vital role in preventing the initiation and progression of obesity. EGCG could cause oxidative stress thus damaging adipocyte directly and activating AMPK and then affecting relative genes and protein expression and signal transduction in various tissues indirectly. However, the increase of oxidative stress in fat accumulation might be an important pathogenic mechanism of obesity-related metabolic syndrome, such as diabetes. Firm conclusions as to whether prooxidant effects of EGCG could perform on body weight, body fat, and adipose weight in humans will require more thorough clinical studies.

### 3.3. Prooxidant and Antibacterial Effects of EGCG

EGCG exhibits a broad spectrum of bactericidal activity against various bacteria. Its bactericidal effects include damage to the bacterial cell membrane and inhibition of fatty acid synthesis and enzymatic activity [55]. H_2_O_2_, which is generated by EGCG, appears to play an indispensable role in the bactericidal actions of EGCG. The bactericidal action of EGCG was related to H_2_O_2_ level, as bactericidal action was inhibited by the increase of CAT concentration [14]. EGCG was found to have bactericidal activity at higher concentrations in the Salmonella assay, highly correlated with H_2_O_2_ production [56]. EGCG showed a dose-dependent (100–1000 *μ*M) inhibition on *Escherichia coli* (*E. coli*) OP50 strain growth [57]. This inhibitory action was associated with a profound increase in intracellular oxidative stress caused by EGCG. Hence, the use of EGCG as a prooxidant is well supported by these studies.

EGCG was shown to have broad antibacterial spectrum effects on both gram-positive and gram-negative bacteria. Nevertheless, EGCG might function through different mechanisms against gram-positive and gram-negative bacteria. Intracellular ROS level was determined by flow cytometry. The results indicated that damage on gram-negative *E. coli* cell walls was induced by EGCG depending on H_2_O_2_ release [58]. In contrast, the damages on gram-positive *Staphylococcus aureus* resulted from a combination between EGCG and peptidoglycan layer [59]. Because the outer membrane of gram-negative bacteria was mainly composed of negatively charged lipopolysaccharides, which could resist the destruction of EGCG, they are less susceptible to EGCG than gram-positive bacteria [57].

Bacterial cell membrane damage not only prevents the binding of bacteria to host cells but also inhibits the ability of the bacteria to combine with each other and form biofilms [55]. EGCG was known to attack the lipid bilayer of bacterial cell membranes, leading to physical disruption of the membrane [59]. As for the cell walls, results from atomic force microscopy suggested that the subminimum inhibitory concentrations of EGCG treatment (60-250 mg/L) to *E. coli* O157:H7 strains could lead to temporary changes in the cell walls (Cui et al., 2012). Such changes were due to the damage caused by H_2_O_2_ generated from EGCG. Moreover, EGCG caused cell membrane damage via increased intracellular ROS level and led to potassium leakage. These are potentially conducive to the antibiofilm efficacy of EGCG against *Vibrio mimicus*, which is a food-borne pathogen in seafood and water [60].

In addition, EGCG also regulates the expression of oxidative stress-related genes. OxyR and SoxRS systems are activated upon exposure to oxidative stress. OxyR induces katG and SoxRS induce sodA strongly when cells are stressed by exogenous H_2_O_2_ [61]. EGCG treatment upregulated katG and sodA expression in *E. coli*. These results verified the role of ROS in EGCG-mediated bacterial inhibition [57]. The Cpx system is thought to control protein homeostasis in the cell envelope. When *E. coli* was exposed to EGCG, apoptosis happened because of ROS formation by the Cpx system [62]. RpoS is a general stress regulator in response to oxidative stress. EGCG could cause a reduction in the expression for RpoS, indicating that EGCG induced oxidative stress in bacterium models [63].

The potential prooxidant properties of EGCG could be attributed, in part, to its suppressive effects on bacteria. More broadly, research is also needed to determine relative signaling pathways and proteomic factors. EGCG is superexcellent natural products; it could increase the efficacy of bactericidal effect when it aids other fungicides. More recent attention has been focused on the impact of green tea and green tea polyphenols on the intestinal microflora. Whether EGCG intervention would change the diversity of microbiota and lead to microbiota death is also in need of further investigation.

## 4. Adverse Effects

In recent years, EGCG has become one of the most aggressively promoted food supplement products in daily life. EGCG entered the market and its safety has raised queries. The prooxidant effect of EGCG is not necessarily advantageous; they might have implications regarding potential toxicity. Hence, it is necessary to systematically explore the harmful effects of EGCG and the mechanisms.

### 4.1. Prooxidant and Hepatotoxicity Effects of EGCG

A considerable amount of literature has been published on hepatotoxicity of green tea-derived products [7]. It is noteworthy that the hepatotoxicity of green tea and its derived products was initially found in some diet products. In 2003, after being the cause of liver injury in 13 subjects, France and Spain governments have suspended the marketing of Exolise, which was a weight-loss phytotherapeutical drug [64]. In the past two decades, reports on liver disorders caused by green tea ingestion with overdose of EGCG content have gradually emerged [65].

The liver is a major drug metabolic organ in the body. The bioavailability of EGCG in rats was determined after 60 min of oral administration (500 mg/kg) by detecting the concentration of EGCG in plasma and different tissues including the liver. The results showed that the concentration of EGCG in the liver (48.4 *μ*mol/kg) was four times higher than in that in the blood plasma (12.3 *μ*mol/kg) [66]. Moreover, utilizing anatomy, EGCG could trigger liver damage, whereas no visible abnormalities were found in other tissues and organs [65, 67]. Hence, it could be preliminarily concluded that the liver is the toxic target organ of EGCG.

At the cellular level, EGCG demonstrated cytotoxic effect in cultured rat hepatocytes. It was shown that 200 *μ*M EGCG treatment on freshly isolated rat hepatocytes caused time-dependent cytotoxicity [68]. The hepatocyte was incubated with EGCG for 24 h, resulting in liver cell function reduced dose dependently [69]. The decrease of lactate dehydrogenase (LDH), a marker of cell membrane damage, was observed in rat hepatocytes [69]. EGCG also caused damage to the outer mitochondrial membrane, by the fact that mitochondrial membrane potential collapsed [68].

In animal experiments (Table 2), the severity of EGCG-induced toxicity is relevant with dose, route of administration, and period of treatment [65, 67, 68, 70–75]. Biochemical and histopathological analysis showed that liver samples of mice displayed different degrees of liver injury. Liver function indexes of plasma alanine aminotransferase (ALT) and aspartate aminotransferase (AST) activity increased in a dose-dependent manner.

Malondialdehyde (MDA) and 4-hydroxynonenal (4-HNE) are final products of lipid peroxidation, present biochemical markers of oxidative stress. Metallothionein (MT) and *γ*-histone 2AX (*γ*H2AX) are molecular markers of oxidative stress. Oral high dose of EGCG (750 mg/kg/d) to CF-1 mice for two days significantly enhanced the formation of MDA in the liver and elevated the expression of hepatic MT and *γ*H2AX protein and increased positive staining for 4-HNE in liver samples [65]. Intraperitoneal administration of EGCG (55 or 75 mg/kg/d) for five days raised serum 4-HNE level, and western blot analysis showed that hepatic *γ*H2AX was markedly increased [71]. All these biomarkers illustrated that EGCG-triggered hepatotoxicity in vivo was induced by oxidative stress.

Previous pharmacological studies have shown that under normal physiological conditions, EGCG is metabolized through methylation, sulfation, and glucuronidation and then excreted in urine subsequently [76], whereas at toxic doses these pathways might be saturated, and the excessive amount of EGCG would be oxidized to form o-quinone, which could react with GSH to form EGCG thiol conjugates [74]. Therefore, it could be inferred that high dose of EGCG results in the accumulation of o-quinones, and the metabolites of o-quinones are biomarkers of oxidative stress. Two EGCG thiol conjugates (EGCG-2′-cysteinyl and EGCG-2′′-cysteinyl) were detected in the pooled 24 h urine of mice treated with a dose of 200 or 400 mg/kg intraperitoneal (i.p.) injection of EGCG. However, EGCG thiol conjugates were not found when the dose was 50 or 2000 mg/kg BW i.p. [74]. When CF-1 mice were treated with a single dose of 1500 mg/kg intragastric (i.g.) administration of EGCG, both EGCG-2′-cysteine and EGCG-2′′-cysteine were detected in the pooled 24 h urine [65]. GSH conjugate of EGCG was also detected in hepatocytes incubated with EGCG [68]. These findings indicated that the formation of detectable amounts of EGCG thiol conjugates appears to result from the administration of toxic doses of EGCG.

Nuclear factor erythroid-related factor 2 (Nrf2), an essential antioxidant transcription factor, regulates the expression of many antioxidant and phase II detoxifying enzyme genes, such as HO-1 and NADP(H):quinone oxidoreductase-1 (NQO1), through antioxidant response element (ARE). Under normal metabolic and physiologic states, Nrf2 is repressed in the cytoplasm by Kelch-like ECH-associated protein-1 (Keap1). While under oxidative stress conditions, Nrf2 dissociates from Keap1 and translocates to the nucleus to bind to ARE. The activation of the Nrf2-ARE signaling pathway, representing a major cellular defense against oxidative stress, could stimulate the expression of downstream antioxidant enzymes [77]. A previous study revealed that toxic doses of EGCG (55 and 75 mg/kg, i.p.) inhibited hepatic antioxidant enzymes (SOD, CAT, and glutathione peroxidase 1) and exacerbated oxidative damage in hepatocytes [71]. After treatment with EGCG, the expression of Nrf2 decreased in the cytosol and increased in the nucleus, indicative of Nrf2 activation. As a result, mRNA expression of HO-1, NQO1, and other hepatic Nrf2-target genes was induced in a dose-dependent manner. Accordingly, a conclusion could be made that the molecular mechanisms underlying high-dose EGCG potential toxicity involve activation of the Nrf2-ARE signaling pathway and suppression of major antioxidant enzymes.

It has been suggested that individuals with low catechol-O-methyl transferase (COMT) activity might be more susceptible to EGCG-induced hepatotoxicity [78]. COMT is a crucial enzyme in protecting cells from EGCG-mediated oxidative stress and hepatotoxicity, and it could convert EGCG to a methylated conjugate [68]. Treatment with dicumarol, an NQO1 inhibitor, was found to cause a significant increase in EGCG cytotoxicity and ROS formation, suggesting that hepatotoxicity caused by EGCG o-quinone metabolites could be reductively detoxified by NQO1 [68]. Studies conducted by Wang et al. demonstrated that melatonin attenuated EGCG-triggered acute liver damage and associated Nrf2 inhibition at the nonlethal dose of EGCG, suggesting that the Nrf2-mediated signaling pathway plays a vital role in counteracting EGCG toxicity [70].

**Figure 5:**
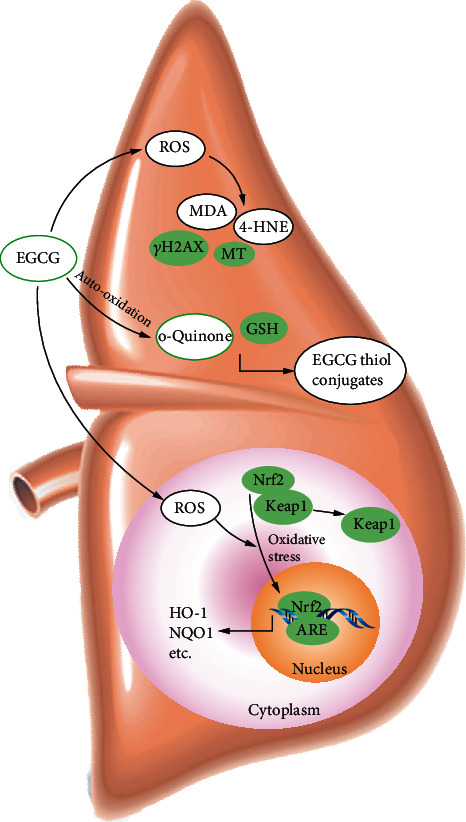
The hepatotoxicity mechanism of EGCG.

The oxidative stress induced by high-dose EGCG is dominated in the liver. The hepatotoxicity mechanism of EGCG involves the formation of lipid peroxidation products and EGCG thiol conjugates, as well as activation of the Nrf2-ARE pathway (Figure 5). The adverse events of hepatotoxicity caused by EGCG also occurred in the population. Clinical manifestation includes elevated serum transaminase and bilirubin levels, abdominal pain, and occasional jaundice [7]. Exploring safer and more efficient natural products and using in combination with EGCG, thereby detoxifying EGCG-induced hepatotoxicity, would be a fruitful area for further work.

### 4.2. Prooxidant and Nephrotoxicity Effects of EGCG

Inoue et al. reported that high-dose EGCG caused nephrotoxicity in dextran sulfate sodium-induced colitis mice, as revealed by increases in serum creatinine, the most reliable biomarker of nephropathy [79]. Moreover, the antioxidant enzymes (HO-1 and NQO1) and heat-shock protein 90 (HSP 90) expressions were downregulated. It can be speculated that high-dose EGCG disrupts kidney functions through the suppression of antioxidant enzymes and heat-shock protein expressions, which might be associated with its prooxidative property.

Since ROS is generated enormously, oxidative stress occurred, and defense systems would be overwhelmed. Several evidences suggest that oxidative stress plays a vital role in the development and progression of diabetes and its related complications [80]. EGCG acts as a prooxidant that might further augment oxidative stress. A previous study indicated that EGCG treatment to streptozotocin- (STZ-) induced diabetic mice would result in a deteriorated oxidative stress status [81].

The kidney is a vital organ affected by diabetes. EGCG treatment (100 mg/kg/d, i.p. for 4 days) enhanced NADPH oxidase contents, while it reduced total antioxidant capacity and downregulated Nrf2, HO-1, and HSP 90 in STZ-induced diabetes mouse kidneys. Histopathological examination confirmed that EGCG could cause kidney damage [81]. In contrast, EGCG might be helpful to inhibit the progression of diabetes through suppressing the increase of blood glucose levels, when the same dose of EGCG (100 mg/kg) and the same administration route (i.p.) were given to STZ-induced diabetic mice for 10 days [82]. These results suggested that EGCG nephrotoxicity could occur even under low dosage. The administration duration might be one of the crucial factors that affect EGCG toxicity. As a result, although many studies have demonstrated the ability of EGCG to mitigate or retard diabetes, patients with diabetes are advised to take EGCG supplements with caution.

### 4.3. Prooxidant Effects and Other Adverse Effects of EGCG

Except the liver and kidney, EGCG-induced adverse actions related to its prooxidant effects were observed in other organs and/or tissues. In animal studies, Chu et al. [83] found that EGCG could give a deleterious effect to ocular tissues, as oxidative stress was induced in the plasma, aqueous humor, vitreous humor, cornea, and retina. High-level EGCG would induce SOD1 but suppress CAT expression, resulting in oxidative stress in the retina. The increase of SOD1 would accelerate ROS neutralization rate to form H_2_O_2_, while the decrease of CAT would retard the rate of H_2_O_2_ clearance.

Oxidative stress could lead to the destruction of islet *β*-cell and decrease the sensitivity of peripheral tissues to insulin, resulting in type 1 diabetes [84]. A 4-day treatment with EGCG (5 mg/kg/d, i.p.) further damaged the *β*-cell response to high glucose in STZ-induced diabetic rats [17]. Antioxidant vitamin E blocked the death and dysfunction of *β*-cell, suggesting that EGCG acted as a prooxidant.

An in vitro study reported the embryonic toxicity of EGCG. 25–50 *μ*M of EGCG notedly increased apoptosis in mouse blastocysts, decreased cell number, and appeared to impair sequent embryonic development [85]. EGCG induced injury in mouse blastocysts through intrinsic apoptotic signaling processes, because pretreatment with specific inhibitors of caspase-9 and caspase-3 effectively blocked apoptosis. This intrinsic apoptosis is related mainly to the prooxidant activities of EGCG.

With respect to cellular DNA, EGCG has obvious damaging activities. In healthy human lymphocytes, 1-100 *μ*M EGCG induced the DNA strand breakage [16]. This finding is consistent with other research that found EGCG (10-100 *μ*M) causes DNA damage in a dose-dependent manner in human lymphocytes [86]. This oxidative damage results from prooxidant effects of EGCG. It is worthy to note that EGCG has no clastogenic effects in vivo [56].

Another in vitro study found that EGCG-induced oxidative stress in fat cells might be detrimental to health by interfering with the adipocyte endocrine activity [87].

## 5. Safe Dose of EGCG

EGCG-based dietary supplements have been considered as healthy and natural products. Although the toxic doses of EGCG are much higher than those delivered by daily tea consumption, they are more readily achievable in the context of dietary supplements. Hence, it is necessary to discuss the safe dose of EGCG.

In a safety study on EGCG, the genetic, acute, and short-term toxicity was examined, and a no-observed adverse effect level (NOAEL) of 500 mg/kg/day EGCG was established [73]. The oral LD_50_ value of EGCG in rats was found to be between 186.8 (safe) and 1868 mg/kg (shows toxic effects and animal morbidity and mortality) [73].

As for humans, the NOAEL was reported to be 600 mg/day [88]. The acceptable daily intake (ADI) for 70 kg adult humans was reported to be 322 mg EGCG/day [88]. Likewise, some European regulatory agencies proposed that the tolerable upper intake level of EGCG should be 300 mg per day for humans [88]. After reviewing the evidence from interventional clinical trials, the European Food Safety Authority (EFSA) concluded that an intake of 800 mg or more of EGCG/day could lead to elevated transaminases [64]. Based on the available data on human adverse event data review, an observed safe level of EGCG might be considered for 704 mg per day in beverage form and 338 mg per day in bolus form [78].

100 g dry weight of green tea contains about 7000 mg EGCG, and 100 g of green tea infusion contains approximately 70 mg EGCG. The mean exposure EGCG from brewed green tea is 321 mg/day in adults [64]. It is challenging to determine a standard safe intake level for EGCG, as the data derived from a large set of human clinical studies might be diverse in designs, durations, and subject populations. Even so, it is important to check the label carefully and calculate the daily intake of EGCG when taking dietary supplements. EGCG intake might require health-based guidance when there are other EGCG sources.

## 6. Conclusion and Perspective

**Figure 6:**
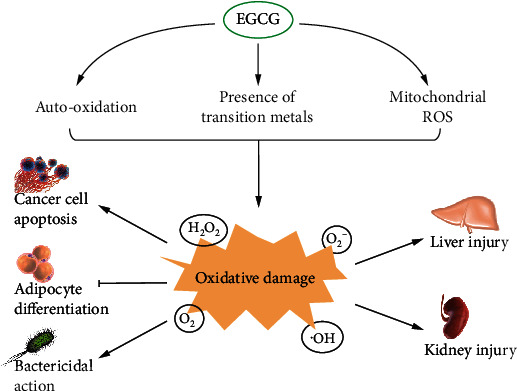
The prooxidant effects of EGCG.

EGCG has a variety of beneficial functions, which could be attributed to its antioxidant properties. However, EGCG could also function as a prooxidant under certain conditions (Figure 6). Its prooxidant effects into the cells were evidenced as oxidative damage to the cell structures, including DNA and lipids. This oxidative damage results from excess ROS produced by EGCG. Several approaches have been proposed to explain how EGCG induces the production of a mass of ROS. They include autooxidation processes of EGCG, presence of transition metals, and mitochondrial ROS. On the one hand, the prooxidant effects of EGCG exhibit salutary effects, namely, induce cancer cell apoptosis, inhibit adipocyte differentiation, and cause bactericidal action. These actions finally result in cell damage or death, including cancer cells, fat cells, and bacterial cells, suggesting that EGCG is a potent cytotoxic agent. On the other hand, the prooxidant nature of EGCG has been questioned, due to the excess intake of EGCG which induced toxicity in animal models and human subjects. High-dose EGCG overproduces ROS, resulting in the damage of antioxidant defense in the body. Local organs, especially the liver, could be involved in injuries to different extents.

According to the gathered data, the oral administration (p.o.) or i.g. is much less toxic than i.p., probably due to the limited systemic bioavailability of oral EGCG. However, under specific conditions, such as fasting and repeated administration, EGCG plasma levels might rise and reach toxic levels [6]. The majority of the toxic effects of EGCG are acute, subacute, and subchronic toxicity. Up to now, far too little attention has been paid to chronic toxicity, which evaluates the toxic effects of EGCG in longer than three months of exposure. Given that consuming green tea and taking supplements are long-term processes, the chronic toxicity of EGCG is an important issue for future research.

Moreover, as the relationship between natural drug metabolism and cytochrome P450 enzymes has gradually been revealed, EGCG might worsen hepatic oxidative stress by influencing drug-metabolizing pathways. The relationship between cytochrome P450 enzyme activation and EGCG metabolism provides an insight for future research on hepatotoxicity mechanism. Cytochrome P450 might interact with EGCG and produce oxygen free radical, thus disrupting mitochondrial membrane potential [89].

Most prooxidant effects of EGCG relevant to health benefit research are based on in vitro studies. There is relatively little clinical trial which might be due to the potential toxicity of high doses of EGCG in human subjects. It is hard to determine when such prooxidant effects transition from beneficial effects to potentially harmful effects. Further research regarding the appropriate doses for human intervention studies would be worthwhile.

The dual function of antioxidant and prooxidant potentials of EGCG is mainly dependent on the dose levels and the biological environments. Some of EGCG-mediated health effects are only affected by concentrations, which are far above the levels obtained by either drinking green tea or taking moderate doses of green tea extract-based dietary supplements. These doses might present to be toxic in some cases. The presence of antioxidants such as melatonin could lower the toxicity of EGCG. Antioxidants might be able to minimize the increase of ROS generation induced by EGCG. Safe and effective food therapeutics searched from natural drugs has provided a new idea for diminishing the potential adverse effects of EGCG. However, the matter of concern is whether a loss of the beneficial effects accompanies the combination of EGCG with other antioxidants. Moreover, due to the Fenton reaction, when transition metal levels are considerably higher, the dosage of EGCG required to elicit prooxidant effects would be significantly lower [42]. Hence, further investigation is needed to optimize the dosage to offer salutary effects with minimal unfavorable effects.

## Figures and Tables

**Table 1:** Role of prooxidant effects in the anticancer activity of EGCG based on cell culture studies.

Cell lines	EGCG concentration	Time	Biological effects	References
Bladder cancer				
NBT-II	10-40 *μ*M	24-72 h	Induced early apoptosis through DNA damage	[18]
Breast cancer				
MCF-7	10-50 *μ*g/mL	48 h	Induced cell growth inhibition and apoptosis by downregulating survivin expression via suppressing the AKT pathway and activating caspase-9	[19]
MCF-7	10-400 *μ*M	6 h	Induced apoptosis at low doses via activation of JNK, caspase-9, and caspase-3, while inducing necrosis at high doses, which is related to differences in ROS generation and ATP levels	[20]
Cervical cancer				
HeLa	50 *μ*M	24 and 48 h	Increased cell death through DNA damage	[21]
HeLa	25-200 *μ*M	1 h	Induced cell death through generation of ROS and inactivation of Trx/TrxR	[22]
Colon cancer				
HCT116	50-100 *μ*M	24 h	Induced apoptosis through induction of ROS and epigenetic modulation of apoptosis-related gene expression	[23]
HT-29	25-250 *μ*M	36 h	Induced apoptotic cell death via activating the JNK pathway, accompanying mitochondrial transmembrane potential transition and cytochrome c release; IC_50_ was ~100 *μ*M	[24]
Endometrial carcinoma				
Ishikawa	25-150 *μ*M	48 h	Induced apoptosis via ROS generation and p38 MAP kinase activation; IC_50_ was 132 *μ*M	[25]
Esophageal cancer				
KYSE 150	20 *μ*M	8 h	Inactivated EGFR by superoxide generated from autooxidation of EGCG	[26]
Lung cancer				
H661 and H1299	20-100 *μ*M	48 h	Displayed strong growth inhibitory effects against lung tumor cell lines; IC_50_ was 20 *μ*M	[27]
H1299	10-50 *μ*M	24 h	Inhibited cell growth through induction of ROS; IC_50_ was 20 *μ*M	[28]
Lymphoblastic leukemia				
Jurkat	12.5-50 *μ*M	6 h	Induced apoptosis via H_2_O_2_ production and hydroxyl radical formation	[29]
Myeloma				
IM9, RPMI8226, and U266	20-100 *μ*M	24-72 h	Induced apoptosis by modifying the redox system	[30]
Oral cancer				
SCC-25 and SCC-9	100 *μ*M	1-6 h	Reduced cell viability by inducing mitochondria-localized ROS and decreasing SIRT3 expression	[31]
Ovarian cancer				
SKOV-3	20-50 *μ*g/mL	2 d	Inhibited cell proliferation and induced apoptosis by inhibiting cell cycle arrest and inducing DNA damage	[32]
Pancreatic cancer				
PANC-1	20-60 *μ*M	12 h	Induced apoptosis through generation of ROS, as well as caspase-3 and caspase-9 activation	[33]
MIA PaCa-2	100-200 *μ*M	24 h	Induced stress signals by damaging mitochondria and ROS-mediated JNK activation	[34]
Primary effusion lymphoma				
BCBL-1 and BC-1	20 *μ*g/mL	24 h	Induced apoptosis and autophagy through ROS generation	[35]
Prostate cancer				
PC3	1 and 25 *μ*M	48 h	Reduced cell survival and increased apoptosis; caused a significant alteration in caspase-9 alternative splicing	[36]

**Table 2:** Hepatotoxicity of EGCG based on animal models.

Animal type	EGCG dosage (mg/kg/d)	Route of administration	Duration	Results	Reference
Female Swiss albino mice	108, 67.8, 21.1, and 6.6	i.p. and p.o.	14 d	i.p. treatment increased serum bilirubin markers; p.o. treatment did not show any dose-dependent changes except ALT marker. 14 d tolerable dose of EGCG was 21.1 mg/kg for i.p. and 67.8 mg/kg for p.o.	[67]
Male Kunming mice	55	i.p.	5 d	Serum ALT, AST, 4-HNE, IL-2, IL-6, and IL-10 and hepatic *γ*H2AX were raised. Hepatic Nrf2-target gene expression was increased.	[70]
	70		2 d	The fatality rate was 100%.	
	125		Single dose	Serum ALT, AST, 4-HNE, IL-6, and IL-10 and hepatic *γ*H2AX were raised. Hepatic nuclear and cytosolic Nrf2 proteins were suppressed.	
Male Kunming mice	45	i.p.	7 d	Mouse growth was not affected. The dosage was considered as maximum tolerable dose.	[71]
	55 and 75		5 d	Hepatotoxicity occurred. Major hepatic antioxidant enzymes were suppressed. Nrf2-mediated rescue response was induced.	
	75, 100, 200, and 400		Single dose	Mice died in a dose-dependent manner.	
	200		4, 12, and 24 h	The Nrf2 pathway was not activated; Nrf2 and its target genes were suppressed.	
Male ND-4 mice	750	i.g.	5 d	ALT was slightly increased. Histopathology of the liver showed congestion of sinusoids and central and portal veins.	[72]
	1500		Single dose	ALT was markedly increased. Histopathology of the liver showed degenerative hepatocytes and a small number of vacuoles.	
Male CF-1 mice	500	i.g.	7 d	Mouse survival was reduced by 30%.	[65]
	750		7 d	Mouse survival was reduced by 75%. Hepatic MDA, MT, and *γ*H2AX were increased.	
	1500		Single dose	ALT was increased by 108-fold. Mouse survival was reduced by 85%. EGCG-2′-cysteine and EGCG-2^″^-cysteine were detected in the urine.	
Wistar rats of both sexes	1868	p.o.	Single dose	Mice were lethargic and their respiration was labored.	[73]
Male CD-1 mice	100, 150, and 300	i.p.	Single dose	Plasma ALT was increased. Mice died within 24 h.	[68]
Mice	50, 200, and 400	i.p.	24 h	EGCG thiol conjugates (EGCG-2′-cysteinyl and EGCG-2^″^-cysteinyl) were detected in the urine.	[74]
Female Swiss-Webster mice	50	i.p.	7 d	67% of mice died. Plasma ALT activity was elevated. Severe hepatic necrosis occurred.	[75]
